# Some Facial Deformities and Prevention.—Abstract

**Published:** 1898-08

**Authors:** A. E. Baldwin


### Some Facial Deformities and Prevention—Abstract.

BY A. E. BALDWIN, M.D., L.L.B., D.D.S.

Read before the Dental Section of the American Medical Association.

Having been a close observer of the human face for more than a score of years, I feel that should I properly present the subject to you, and that through you and the readers of our most able journal, humanity at large may be much benefited in several ways.

In the first place, it is proper to say that we seldom see a perfectly developed human frame, or a frame even entirely free from deformity, and this truth is even more true of the face than of almost any other part of the frame. To some it may come as a surprise, that it is a very rare thing to find a person, the two sides of whose jaws (either upper or lower) are alike.

In the growth or development of the child into the adult, the head, as a whole, grows much less than the body or extremities, but the growth of the jaw in the normal person is relatively great, even at the age of two or three years, and with the full complement of twenty deciduous teeth the jaws are abnormally small for the proper contour of the bony outlines of the face. But this lack of contour is not noticeable, for the face of the little child is, by the deposit of soft tissue, kept in the "dumpling" shape, but as the child approaches adult life and nature enlarges the jaw, the soft tissue is re-absorbed and the bony outline appears more distinctly, either normal or abnormal in size, making the perfect or the deformed face, as the case may be.

Now, a few words about the normal change from the deciduous to the permanent set. About the sixth year the jaw is surprised by the appearance (at the rear of each of the second deciduous molars) through the gums of the largest, strongest and best-rooted teeth that are given to man—these, as you know, are called the first permanent molars, coming in as they do at the rear of the deciduous teeth they act as abutments for the anterior arch in its development, as the jaw between these two points must, in a few years, (four to seven) appear ten teeth of nearly double the size of the teeth to be replaced, at least three-fifths more space is needed for the regular eruption of these ten permanent teeth than was needed by the deciduous. These abutment teeth so deeply and strongly set in the jaws are so placed that they can hardly be moved back by any force applied, and it is evident that nature thus wisely provided that no infringement should be made upon the space to be developed in the rear of these teeth to be occupied by the second and third molar teeth. So you see that to provide for the occupancy in the jaw of the permanent anterior teeth—the jaw must expand. It is a matter of common observation that the newly erupted, permanent, central incisors appear surprisingly large as compared with the old—being formed to fit the face for years to come, and often these teeth appear crowded and are quite irregular, often over-lapping or turned edgewise. Some notable exceptions are noticed in cases, where, prior to the eruption of the permanent teeth, you may notice that the deciduous teeth which previously were close together now, at the fourth and sixth year, are growing apart so that the spaces are noticeable and marked. This, when it occurs, is the result of the new wide teeth approaching the surface directly side by side—and they are already getting in their work of spreading the arch. In these cases of the teeth coming in over-lapping or crooked, you are often importuned by the parents who call upon you to help the children to regulate the teeth by extracting the teeth on either side to allow them to come down regularly, as they say. Following the thought of the paper you will see that in the effort to relieve the child you have curbed the expansion of the jaw by removing the cause. This expansion is most largely brought about by the expanding pressure of the incoming larger teeth. Perhaps here is as good a place to present to you a rule almost without an exception, which is of the greatest importance to you in serving the best interests of your little patient. The rule is, "Never extract or recommend the extraction of any deciduous tooth, save to make way for the permanent tooth which is to take its place." For I am sure, by closely adhering to this rule, you will always utilize the forces of nature to produce the necessary expansion of the jaw to natural conformity of the face.

To illustrate this farther and to further emphasize this rule, we will take a case of incoming permanent central incisors in this illustrative case: they are so crowded that they overlap very much and the thoughtless physician, in his supposed kindness to the patient, has removed the deciduous lateral under the careless supposition that it was necessary. In this case the teeth will straighten very quickly, but instead of the expansion of the jaw which would have taken place were the laterals left, the jaw will expand but very little if at all, so the deformity of the face will be marked when the teeth are all extracted.

While on this subject it will not be amiss to speak of the necessity of caring for and preserving the temporary molars. Not only for the purpose of preserving the space for the incoming bicuspids or premolars, by which they are replaced about the eleventh or twelfth year, but also for hygienic reasons, and notably among them may be mentioned the fact that the child, up to the eruption of the bicuspids, needs a perfect masticatory apparatus, even more than the adult, for in the adult the masticatory apparatus need only be used enough to produce for the system pabulum enough to furnish nourishment to make up for the daily waste in the tissue, and in the child, while the waste is greater than in the adult, must be replaced, but there also must be nourishment furnished for the development or growth of all the organs of the expanding and rapidly growing physical frame.

Still another reason for bestowing much care on these teeth is that the child is forming habits for life, and if his teeth are neglected so as to become sore or painfully sensitive to mastication the child will only masticate enough to enable him to swallow the bolus thus so imperfectly divided, and in this way not only forming the habit (too prevalent with Americans, young and old,) of bolting the food; but also, which is even more serious, paving the way for all the ills, nervous and otherwise, that come from an overloaded and overworked stomach.

The writer does not want to claim that all of the deformities of the lower part of the face can thus be prevented, for he realizes that there are also the very powerful factors of inheritance which must also be taken into account; but a proper heeding of these matters by the medical advisor will result in a great lessening of such deformities, and, incidentally, of improving the health and nourishment of the patient while young, and reaching far into age as well.

Some may say that it is improbable that such slight force as the incoming teeth may exert would produce such expansion as occurs in the bony expansion or development of the jaws. It is only necessary to say to such, that in all nature it is seen that the feeble though constant force is much the most powerful source in changing from the normal to the abnormal, or in the growth and development in the normal.

These illustrations in nature are on every hand, and are much easier understood than can be that of the elongation of the long bones of the leg, which lengthening occurs by deposit between the shaft and the articulating surfaces.

In the short time at the disposal of the writer it is only possible to go into anything but generalities, and his short paper has subserved its purpose if it results in the medical profession guarding with much more care the temporary teeth of the little ones, as to the care to be bestowed upon them in keeping them healthy and in seeing that they are retained until the new tooth which is to replace it asks for the position.

